# Optimized border irrigation delays winter wheat flag leaf senescence and promotes grain filling

**DOI:** 10.3389/fpls.2023.1051323

**Published:** 2023-02-13

**Authors:** Feilong Yan, Zhenwen Yu, Yu Shi

**Affiliations:** National Key Laboratory of Crop Biology, Agronomy College of Shandong Agricultural University, Tai’an, Shandong, China

**Keywords:** border irrigation, chlorophyll fluorescence, antioxidant enzyme activity, grain filling, grain yield

## Abstract

Border irrigation is still the main irrigation method in the Huang-Huai-Hai Plain of China (HPC), and the suitable irrigation border length for water saving and high yield under traditional irrigation is still unclear. Therefore, a 2-year traditional border irrigation experiment (2017–2019) was conducted on the HPC. Four border lengths were tested: 20 m (L20), 30 m (L30), 40 m (L40), and 50 m (L50). These treatments were given supplementary irrigation at jointing and anthesis. An exclusively rainfed condition formed the control treatment. Compared with other treatments, the activities of superoxide dismutase antioxidant and sucrose phosphate synthetase, and the contents of sucrose and soluble proteins after anthesis were higher in the L40 and L50 treatments, while the content of malondialdehyde content was lower. Therefore, the L40 treatment effectively delayed the decrease in the soil plant analysis development (SPAD) value and chlorophyll fluorescence characteristics, promoted grain filling, and achieved the highest thousand-grain weight. Compared with the L40 treatment, the grain yields of the L20 and L30 treatment were significantly reduced, while the water productivity of the L50 treatment was significantly reduced. These findings suggest that 40 m was the optimal border length for both high yield and water saving in this experiment. This study provides a simple and low-cost water-saving irrigation method for winter wheat in the HPC under traditional irrigation, which can help alleviate the pressure of agricultural water use.

## Introduction

The Huang-Huai-Hai Plain of China (HPC) is the country’s main wheat cultivation area, accounting for approximately 70% of Chinese winter wheat production; however, this agricultural area has access to only 7% of Chinese total water resources (Fan et al., 2019). In this region, evapotranspiration is approximately 400–500 mm during the winter wheat growing season, while the rainfall in the wheat growing season is only about 100–180 mm (Guo et al., 2014; Yuan et al., 2015). Therefore, supplementary irrigation is the foremost measure that ensures wheat yield. At present, with the intensification of competition among various sectors of social production, the proportion of agricultural water consumption in China has dropped from 97% in the early days of the founding of the People’s Republic of China to 64% (Mo et al., 2016). This situation urgently requires the determination of reasonable irrigation methods and improved agricultural water use efficiency to maximize the lower consumption of water resources. Many researchers have proposed methods to improve crop water use efficiency, including reduced tillage or no-tillage mulch, which increases soil water content but substantially reduces land use, and drip irrigation or sprinkler irrigation, which is used to reduce irrigation water and wheat water consumption but is difficult to promote because of its complicated operation and high cost (Mostafa et al., 2018; Jha et al., 2019). Therefore, the study of traditional border irrigation water-saving technology is important, as this approach could reduce agricultural irrigation water and promote sustainable agricultural development in the HPC.

Adequate soil water is usually considered the primary requirement for ensuring a high wheat yield (Li et al, 2019a). Studies have shown that wheat yield can be guaranteed by irrigation during the jointing and anthesis stages, which are two critical water-requiring stages of wheat development (Xu et al., 2018; Yang et al., 2018). However, unreasonable irrigation measures applied to wheat will lead not only to lower yields but also to wastage of water resources and aggravation of the decline in groundwater levels (Liu et al., 2020b). There are a large number of farmers who irrigate three times or more during the wheat growing season, where each irrigation exceeds 60 mm (Sun et al., 2011). Excessive irrigation is known to reduce the utilization of soil water and precipitation by wheat, thereby reducing water use efficiency (Wang et al., 2018). However, more than 80% of irrigation on the HPC is still border irrigation, as this traditional approach to irrigation is simple and easy to implement (Liu et al., 2013). Yang et al. (2009) found that when the border length of a field is >80 m, a single irrigation volume can reach deeper than 100 mm in the soil, which far exceeds the water required for wheat growth. A survey on the length of irrigation borders in Huimin county, Shandong province, revealed that the border lengths of 87% of the irrigated fields were longer than 100 m, and a border of 180 m increased 40 mm of irrigation compared with a border of 90 m, without an accompanying increase in grain yield (Cui et al., 2006). Our investigation of the plots around the trial sites revealed that more than 97% of the plots had a border length of over 60 m, with some being longer than 200 m. Therefore, it is necessary to conduct field trials to determine the appropriate border lengths of irrigated fields to reduce irrigation water consumption.

Leaf senescence is the final process of leaf development and involves the degradation of macromolecules and the accumulation of reactive oxygen species (ROS) (Nehe et al., 2020). As leaves are the main organs of photosynthesis in wheat plants, premature leaf senescence will reduce the photosynthetic area and shorten the duration of photosynthesis, resulting in an insufficient supply of photosynthates for grain filling, thereby reducing wheat yield (Lv et al., 2017). Soil water is an important factor in regulating antioxidant enzyme activity and affecting leaf senescence (Wang et al., 2013). Consequently, water stress during grain filling substantially reduces the ability of the antioxidant system to remove active oxygen, accelerating the senescence of wheat leaves and resulting in reduced grain yield (Yang et al., 2004; Singh et al., 2012). However, excessive water content does not improve the antioxidant capacity of wheat plants, as shown by Hameed et al. (2011), who found that the catalase activity of flag leaf treated with 100% field capacity was lower than that treated with 75% field capacity. Therefore, exploring the antioxidant capacity of leaves under different border length irrigation is crucial for optimizing the irrigation border length.

Despite the known link between water use efficiency and leaf senescence in wheat, the effect of different border length irrigation on wheat leaf senescence and grain filling has not yet been reported. Therefore, we conducted a field experiment, the objectives of which were to (1) study the fluorescence parameters and antioxidant capacity of flag leaves after anthesis under different border length irrigation, (2) analyze and compare the grouting parameters and grain weight under different border length irrigation, and (3) determine the optimal irrigation border length for high yield and water saving under traditional border irrigation conditions. This study is of great significance for optimizing traditional border irrigation, reducing irrigation water consumption, and improving water use efficiency of winter wheat.

## Materials and methods

### Experimental site

**Figure 1:**
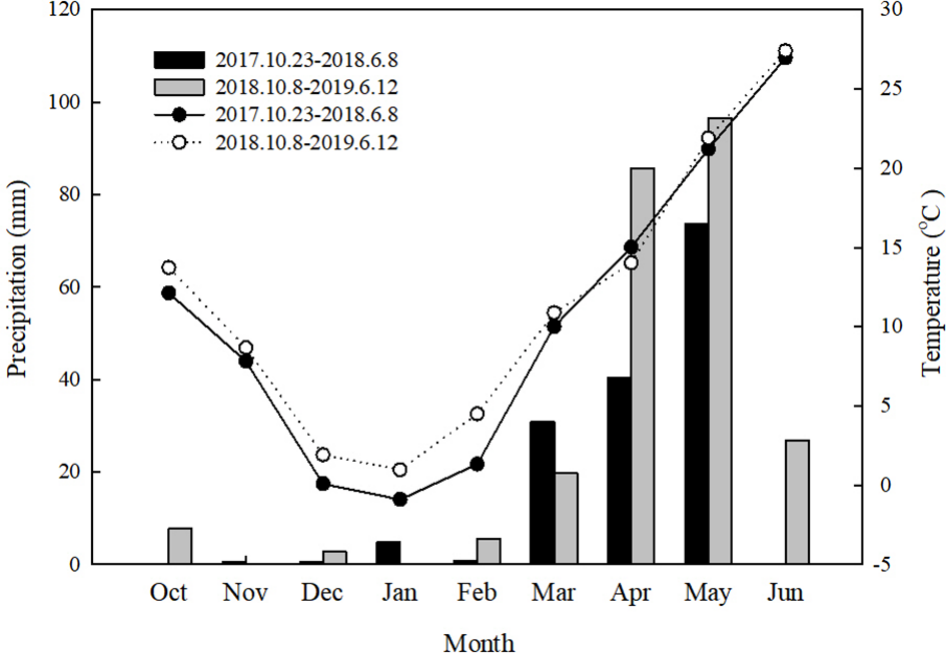
Precipitation and temperature during wheat growth period.

The experimental site was the Shijiawangzi Village, Yanzhou, Shandong province, China (35°42′ N, 116°41′ E), which has a warm temperate semi-humid continental monsoon climate. The soil type in the experimental field is a light loam, and the groundwater level is 25 m deep. The experimental site has an altitude of 49 m and a 1% southeast slope. The field capacity and wilting point of the topsoil were 28.65% and 8.3%, respectively. The precipitation amounts and temperature at different wheat growth stages during the field experiment are shown in **Figure 1**. The nutrient content of the 0–20-cm soil layer before sowing is shown in **Table 1**.

**Table 1:** The nutrient content in the 0–20-cm soil layer before sowing.

Items	Growing season	
	2017–2018	2018–2019
Soil organic matter(g kg^−1^)	14.31	14.24
Total nitrogen (g kg^−1^)	1.17	1.09
Available nitrogen (mg kg^−1^)	118.82	117.32
Available phosphorus (mg kg^−1^)	39.29	36.71
Available potassium (mg kg^−1^)	116.37	122.18

### Experimental design and crop management

**Figure 2:**
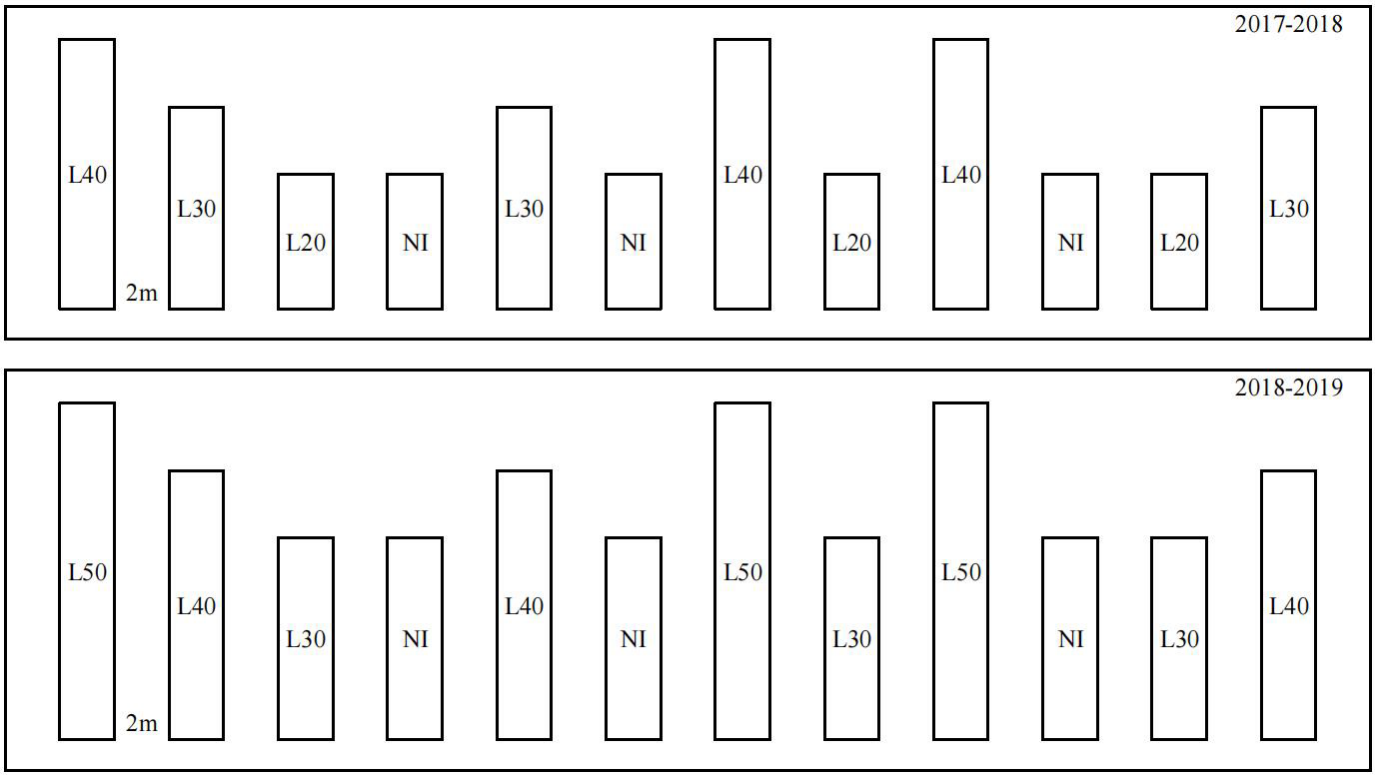
The field layout of experimental treatments.

The experimental setup comprised of different border length irrigation treatments (border width, 2 m) of 20 m (L20), 30 m (L30), and 40 m (L40) in 2017–2018, and 30 m (L30), 40 m (L40), and 50 m (L50) in 2018–2019. A control field that received no irrigation (NI) was set up in each growing season. The treatments were randomly grouped, with three replicates per treatment. A 2-m-wide guard row was used to minimize the effects of water permeating between two adjacent irrigation plots. The field layout of experimental treatments is shown in **Figure 2**. All irrigation treatment fields were irrigated from the same side during the jointing and anthesis stages of wheat growth. The inflow cutoff was designated as 90% (i.e., the irrigation was stopped when the water front reached 90% of the length of the border) (Ji et al., 2014), and the irrigation amount was recorded using a water meter. The water output of the well at the experiment site was 30 m³ h^−1^, and the amount of irrigation during the two growing seasons is shown in **Table 2**. The soil water content of the 0–80-cm soil layer after irrigation is shown in **Supplementary Table S1**.

**Table 2:** The amount of irrigation under different treatments.

Year	Treatment	Jointing (mm)	Anthesis (mm)	Total (mm)
2017	NI	–	–	–
	L20	65.22	54.62	119.84
	L30	77.83	61.17	139
	L40	86.59	69.38	155.97
2018	NI	–	–	–
	L30	63.17	58.11	121.28
	L40	73.02	68.05	141.07
	L50	85.27	79.11	164.38

"-" indicates no irrigated water.

The high-yielding wheat variety “Jimai 22,” the most widely cultivated commercial variety on the HPC, was used for this experiment. The previous crop was summer maize, and the straw was returned to the field after harvest. The experimental plots were planted after twice rotary tillage. Two growing seasons were not irrigated before sowing because soil water content was suitable for sowing. Before sowing, 105 N kg·ha^−1^ (urea), 150 P_2_O_5_ kg·ha^−1^ (superphosphate), and 150 K_2_O kg·ha^−1^ (potassium chloride) were applied as basal fertilizers on all fields, and a topdressing of 135 N kg·ha^−1^ (urea) was applied at the jointing stage. The wheat was sown on 20 October 2017 and 8 October 2018, with planting densities of 270 plants m^−2^ and 180 plants m^−2^, respectively, and harvested on 7 June 2018 and 12 June 2019, respectively. No pests or diseases occurred during the test period.

### Sampling point

In order to reduce errors in the measurement results between treatments, each border length was divided into 10-m intervals, and samples were taken from the center of each interval. The test results are the measured values of the mixed samples at each sampling point under each treatment.

### SPAD value and chlorophyll fluorescence parameters

The soil plant analysis development (SPAD) value of flag leaves was scored using a SPAD meter (SPAD-502, Minolta Camera Co., Osaka, Japan). Measurements from 30 flag leaves with consistent growth were collected at anthesis and 7, 14, 21, and 28 days after anthesis (DAA).

At anthesis and 7, 14, 21, and 28 DAA, five flag leaves per sampling point were measured using an FMS-2 pulse modulated fluorometer (Hansatech Instruments Ltd., King’s Lynn, Norfolk, UK) between 9 and 12:00 a.m. on a sunny and windless day. The leaves were fixed using clamps, and the light-adapted fluorescence parameters were measured first, followed by measurement of the dark-adapted fluorescence parameters after 30 min of dark adaptation. The maximal photochemical efficiency (Fv/Fm), actual photochemical efficiency (ΦPSII), and photochemical quenching coefficient (Qp) were calculated according to Zivcak et al. (2013).

### Sucrose content, and sucrose phosphate synthetase activity and antioxidant capacity determination

A total of 20 fresh samples of flag leaves were collected from each point at anthesis and 7, 14, 21, and 28 DAA. Fresh samples were immediately immersed in liquid nitrogen and then stored at −80°C. The sucrose content and sucrose phosphate synthase (SPS) activity were determined following Feng’s method (Feng et al., 2019). The superoxide dismutase (SOD) activity, soluble protein (SP) content, and malondialdehyde (MDA) content were determined using the method described by Xue et al. (2008).

### Grain-filling characteristics

Wheat spikes that flowered on the same day were tagged, and 20 marked ears per point were harvested at 7-day intervals after anthesis. The samples were dried at 105°C for 10 min and at 70°C to constant weight, and then, the kernels were carefully peeled off the ears. The grains collected from each section in each treatment field were mixed uniformly, and 1,000 grains were randomly counted (three times) using a counting plate and then weighed. The grain-filling process was assessed using the logistic growth equation. For the analysis, we adopted the following secondary parameters to describe the filling characteristics: Tmax (time taken to reach the maximum filling rate), Vmax (maximum filling rate), Vmean (average accumulation rate), and D (grain-filling duration).

### Grain yield and water productivity

Grain yield was determined from a 3-m^2^ quadrat sampled at each point and reported on the basis of 12.5% water content, and yield components were evaluated by the method of He et al. (2020).

Evapotranspiration (ET) was calculated using the following soil water balance equation (Li et al., 2019a): ET = irrigation + precipitation + soil water consumption. Before sowing and at maturity, soil samples of 0–200 cm were taken for each 20-cm layer, and the soil mass water content was determined by drying method. The soil water consumption was calculated using the soil water content measured during the sowing and maturity period. For this experimental site, groundwater recharge and runoff can be ignored.

The water productivity (WP) was defined as WP = GY/ET, where GY is the grain yield and ET is the evapotranspiration (Wang et al., 2013).

### Statistical analysis

The SPSS Statistics 22.0 software (IBM, Armonk, NY, USA) was used to analyze the data, and the least significant difference test (α = 0.05) was used to compare the differences between different treatments. A logistic equation of grain filling was modeled using SPSS 13.0. All charts were generated using Excel and SigmaPlot 12.0 (Systat Software Inc., San Jose, CA, USA).

## Results

### Grain yield and water productivity

The grain yield, yield composition, and WP of the NI treatment were significantly lower than those of the irrigation treatment in both growing seasons (**Table 3**). Different border irrigation had no significant effect on spike number and kernel number. Furthermore, both TWG and grain yield were higher in the L40 and L50 treatments, while the WP was highest in the L40 treatment, which was 3.98%, 4.54%, and 7.94% higher than in L50, L30, and L20 treatments, respectively.

**Table 3:** Grain yield, yield composition, and WP under different treatments.

Year	Treatment	Spike number (*10^4^ ha^−1^)	Kernel number (kernels spike^−1^)	TGW (g)	Grain yield (kg ha^−1^)	WP (kg ha^−1^ mm^−1^)
2017–2018	NI	520.75b	35.3b	33.35d	5,264.18d	16.62d
	L20	563.90a	38.4a	38.88c	7,298.93c	17.76c
	L30	573.26a	38.1a	40.27b	7,656.92b	18.41b
	L40	581.42a	37.9a	41.72a	7,994.25a	19.17a
2018–2019	NI	541.65b	35.4b	35.32c	5,774.47c	14.68c
	L30	594.23a	40.5a	39.78b	8,246.76b	17.20b
	L40	604.78a	40.3a	41.82a	8,762.44a	18.05a
	L50	609.40a	40.2a	41.19a	8,689.07a	17.36ab

TGW, thousand grain weight; WP, water productivity. Different letters indicate significant statistical differences between treatments (p< 0.05). * meaning significant at the 0.05 probability level.

### Antioxidant indices of flag leaves

**Figure 3:**
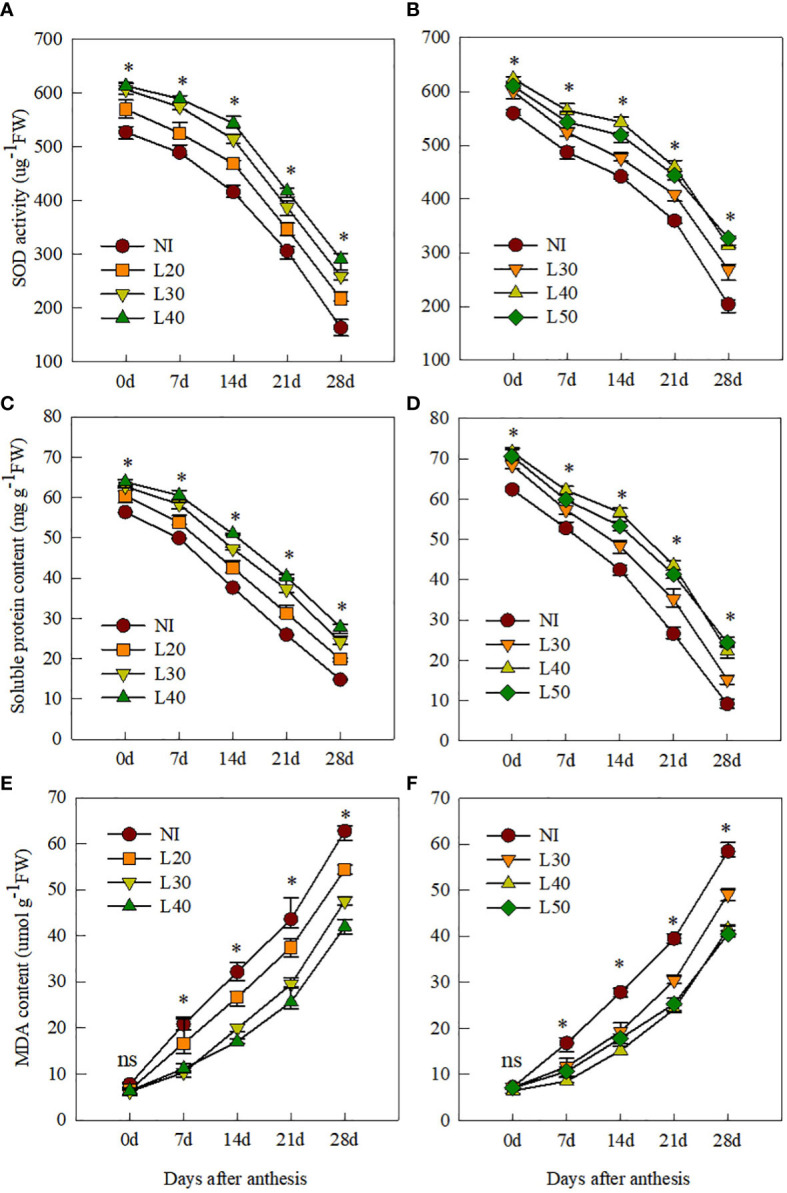
The SOD activity, soluble protein content and MDA content of flag leaves after anthesis under different treatments in 2017-2018 **(A, C, E)** and 2018-2019 **(B, D, F)**. ns, not significant; *P<0.05.

After anthesis, SOD activity and SP content of the flag leaves of all treatments decreased gradually as senescence progressed, whereas the MDA content increased gradually (**Figure 3**). The antioxidant capacity of the NI treatment was significantly lower than that of the irrigation treatment. We found that the treatment with higher irrigation can delay leaf senescence, as there was no significant difference in SOD activity and SP and MDA content among irrigation treatments at 0 DAA; however, from 14 to 28 DAA, both SOD activity and SP content were higher in the L40 and L50 treatments, followed by the L30 and L20 treatments, whereas MDA content in the L40 and L50 treatments was significantly lower than that in the other irrigation treatments.

### SPAD value and chlorophyll fluorescence parameters

**Figure 4:**
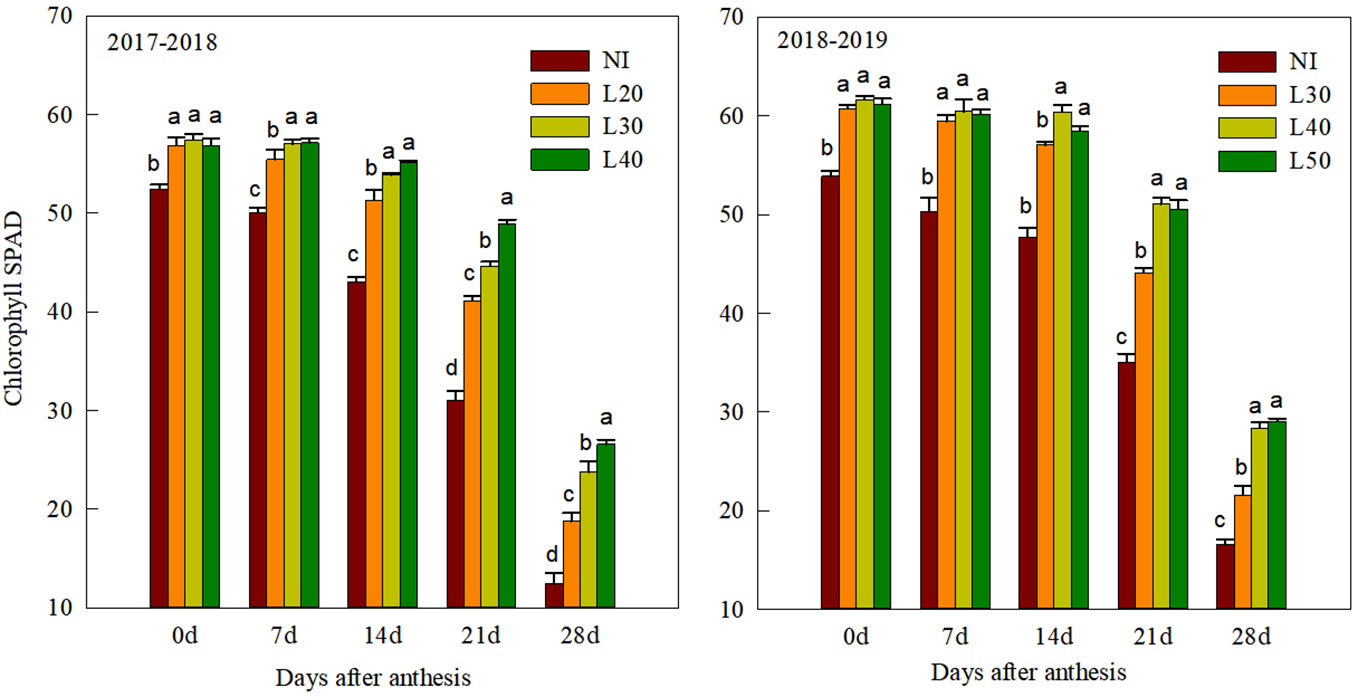
Chlorophyll SPAD value of flag leaves after anthesis under different treatments. Values followed by different letters within the same stages are significantly different at P<0.05.

Compared to the irrigation treatment, the SPAD value of flag leaves in the NI treatment was significantly lower after anthesis in both growing seasons (**Figure 4**). There were no significant differences between the L30, L40, and L50 treatments at 0–7 DAA. The SPAD value of the L40 and L50 treatments showed no significant difference but were significantly higher than those of the L30 and L20 treatments from 14 to 28 DAA.

**Figure 5:**
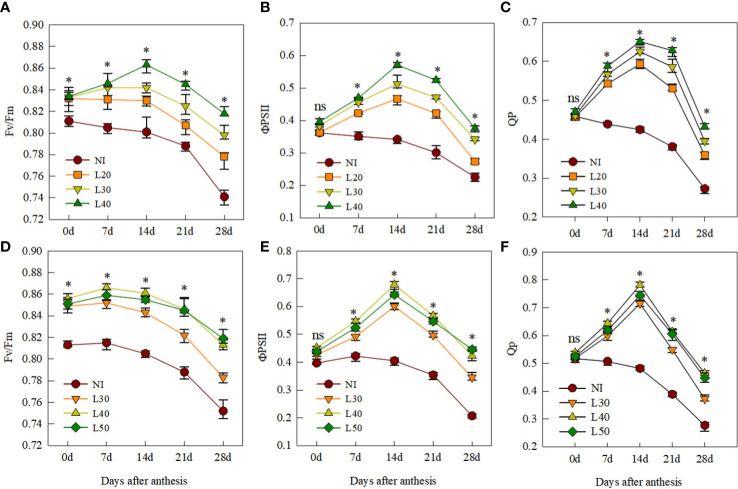
Fluorescence characteristics of flag leaves after anthesis under different treatments in 2017-2018 **(A, B, C)** and 2018-2019 **(D, E, F)**. ns, not significant; *P<0.05.

The changes in chlorophyll fluorescence parameters of flag leaves after anthesis are shown in **Figure 5**. Compared to the NI treatment, Fv/Fm, ΦPSII, and Qp of the irrigated treatments were significantly higher after anthesis. We found that the Fv/Fm, ΦPSII, and Qp of each irrigated treatment showed no significant differences at 0–7 DAA, whereas from 14 to 28 DAA, the Fv/Fm, ΦPSII, and Qp of the L40 and L50 treatments showed no significant differences, which were both significantly higher than those of the L30 and L20 treatments in both growing seasons.

### Sucrose content and sucrose phosphate synthase activity

**Figure 6:**
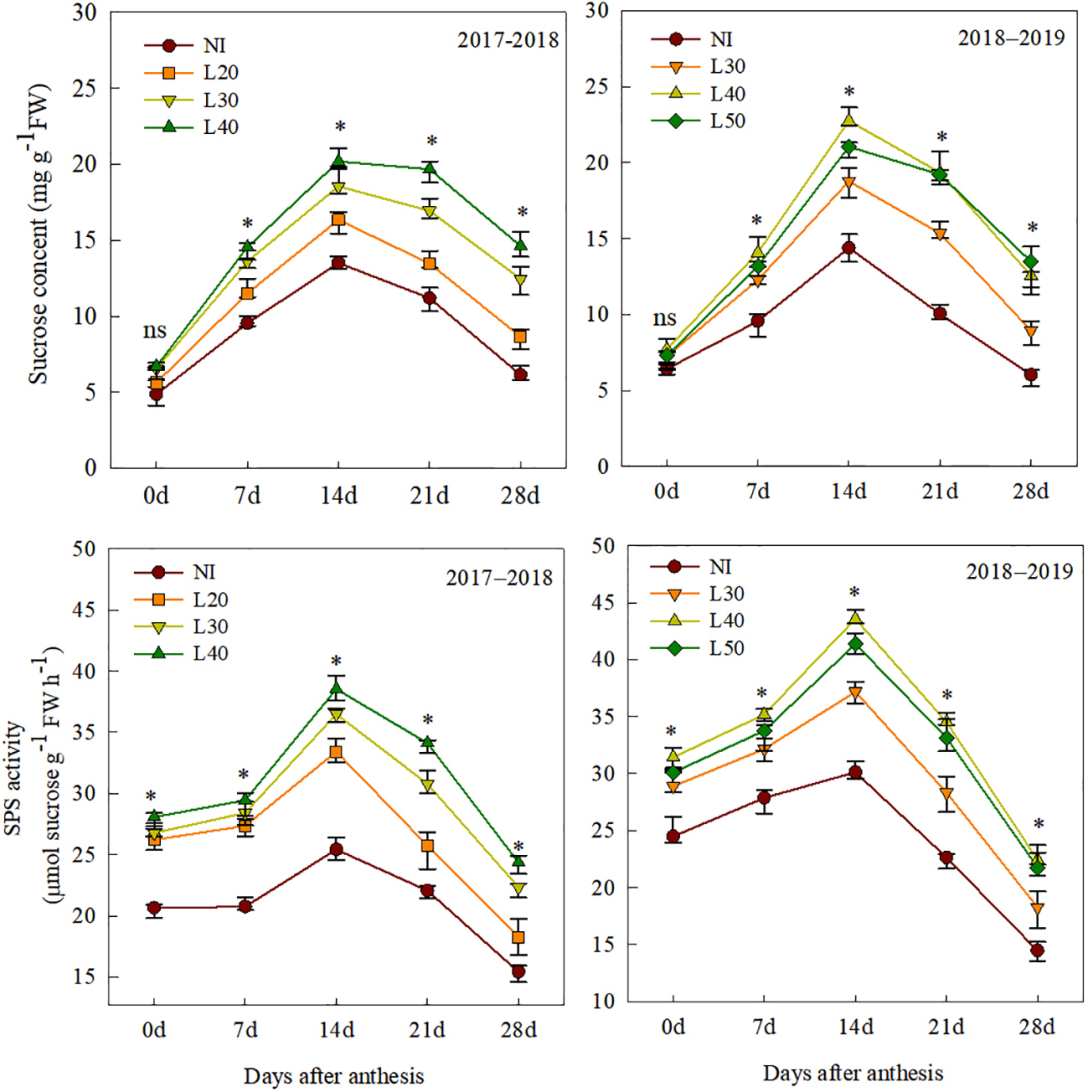
Sucrose content and sucrose phosphate synthase activity of flag leaves after anthesis under different treatments. ns, not significant; *P<0.05.

The sucrose content and SPS activity of flag leaves among all treatments were increased initially and then decreased after anthesis (**Figure 6**). Compared to the irrigation treatments, the sucrose content and SPS activity of the NI treatment were significantly lower. Moreover, different border lengths irrigation had a significant effect on sucrose content and SPS activity. From 14 to 28 DAA, the sucrose content and SPS activity of the L40 and L50 treatments were significantly higher than those of the L30 and L20 treatments, and the sucrose content and SPS activity of the L40 treatment were significantly higher than that of the L50 treatment at 14 DAA.

### Grain-filling characteristics

**Figure 7:**
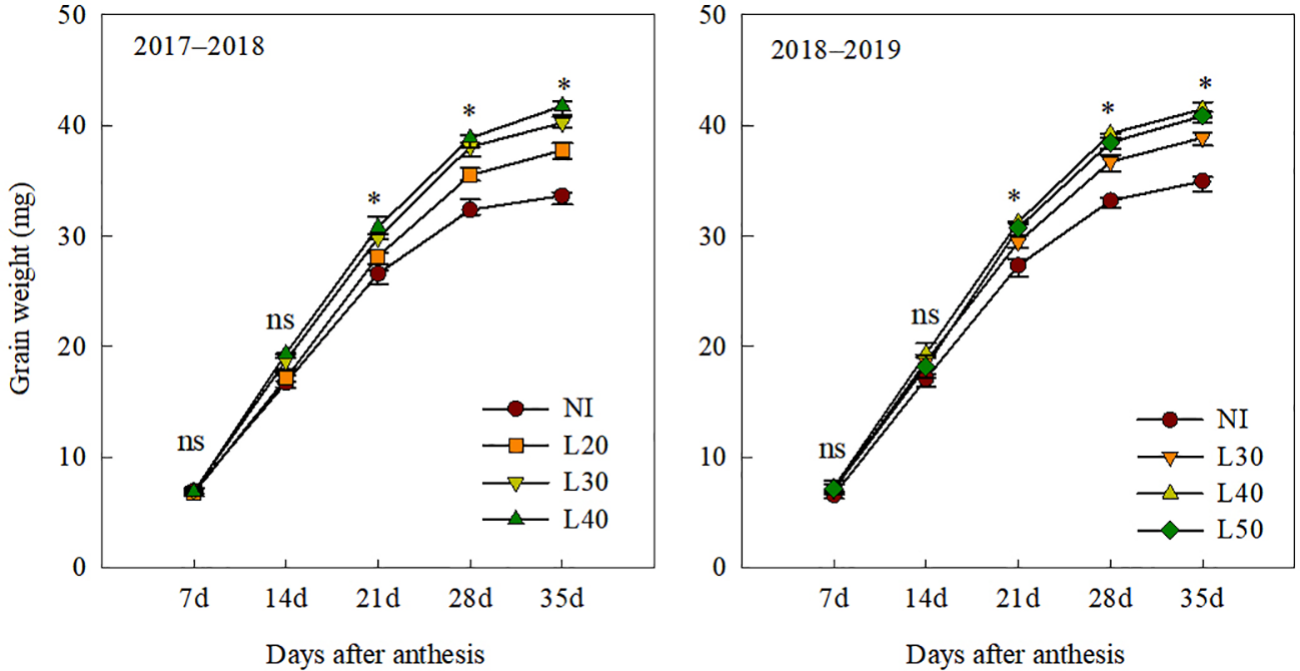
Grain weight after anthesis under different treatments. ns, not significant; *P<0.05.

After anthesis, the grain weight gradually increased with the filling process, and the NI treatment showed significantly reduced grain weight compared with the other irrigation treatments at 35 DAA (**Figure 7**). There was no significant difference in grain weight between all treatments at 7 and 14 DAA. At 21, 28, and 35 DAA, the grain weight was higher in the L40 and L50 treatments, followed by the L30 and L20 treatments. The grain weight of L40 treatment was the highest, which had no significant difference with L50 treatment, which was 4.95% and 10.3% higher than L30 and L20 treatments, respectively.

The grain-filling fitting equations and parameters of each treatment are shown in **Table 4**. There was no significant difference between Tmax and D for all treatments. Both Vmax and Vmean of the L40 and L50 treatments were no significant difference. In addition, in 2017–2018, the Vmax and Vmean of L40 treatment were 3.14%, 3.31%, and 5.91%, 6.61% higher than those of the L30 and L20 treatments, respectively; in 2018-2019, the Vmax and Vmean of L40 and L50 treatments were 6.48%, 5.94%, and 4.86%, 3.48% higher than those of the L30 treatment. Correlation analysis revealed that the grain weight was positively correlated with Tmax, Vmax, and Vmean, but was not correlated with the grain-filling duration (**Table 5**). This indicated that suitable border length irrigation primarily increased grain weight by increasing the grain-filling rate.

**Table 4:** Grain filling equation and grouting parameters under different treatments. .

Year	Treatment	Growth curve equation	Tmax (day)	Vmax (mg grain^−1^)	Vmean (mg grain^−1^ day^−1^)	D (day)
2017–2018	NI	34.15/(1 + 14.97e^−0.19x^)	14.25a	1.62c	0.87c	31.59a
	L20	39.64/(1 + 17.89e^−0.19x^)	15.39a	1.86b	0.97b	32.03a
	L30	41.38/(1 + 17.62e^−0.19x^)	15.49a	1.91ab	0.10ab	32.40a
	L40	42.69/(1 + 17.19e^−0.18x^)	15.41a	1.97a	1.03a	32.49a
2018–2019	NI	36.32/(1 + 15.06e^−0.18x^)	14.75a	1.67c	0.89c	32.63a
	L30	40.53/(1 + 16.24e^−0.18x^)	15.25a	1.85b	0.98b	32.82a
	L40	42.94/(1 + 16.73e^−0.18x^)	15.35a	1.97a	1.04a	32.69a
	L50	42.30/(1 + 17.46e^−0.18x^)	15.61a	1.94a	1.01ab	32.75a

Tmax, the time to reach the maximum filling rate; Vmax, maximum filling rate; Vmean, the average accumulation rate; D, grain filling duration. Different letters indicate significant statistical differences between treatments (p<0.05).

**Table 5:** Correlation analysis between TWG and grain-filling parameters.

	TGW	Tmax	Vmax	Vmean	D
TGW	–	0.84	0.97	0.99	0.62
Tmax	**	–	0.93	0.9	0.61
Vmax	**	**	–	0.98	0.56
Vmean	**	**	**	–	0.58
D	ns	ns	ns	ns	–

TGW, thousand grain weight; Tmax, the time to reach the maximum filling rate; Vmax, maximum filling rate; Vmean, the average accumulation rate; D, grain filling duration. ns, not significant; **p<0.01. "-" indicate that the same indicators cannot be analyzed.

### Correlation analysis

**Figure 8:**
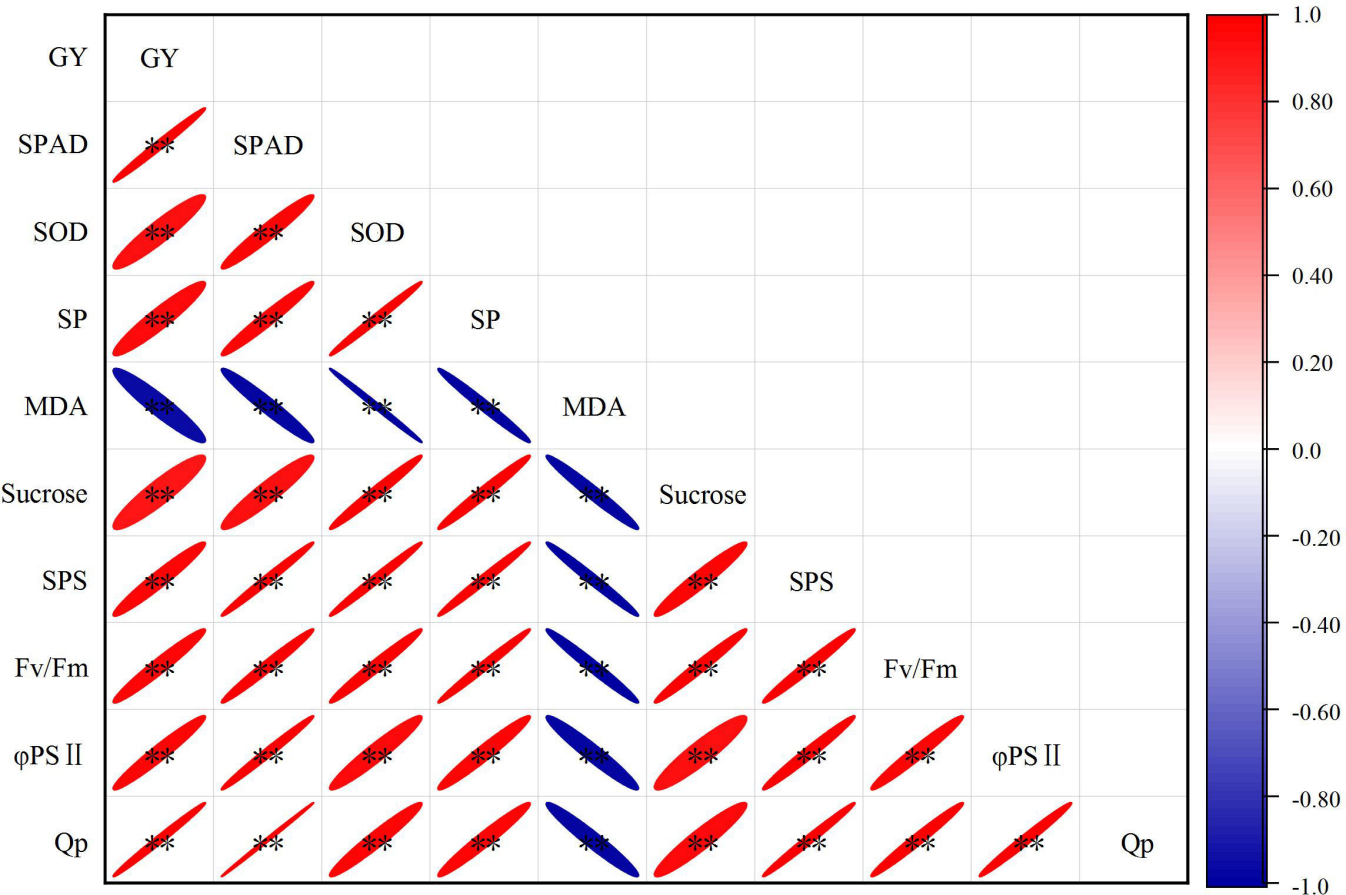
Correlation analysis of grain yield and physiological traits from 2017 to 2019 growing seasons. **, significant at the 0.01 probability level.

**Figure 8** shows the correlation analysis of grain yield, leaf fluorescence parameters, leaf senescence parameters, and grain-filling parameters. This analysis revealed that grain yield was significantly negatively correlated with MDA, but significantly positively correlated with the other measured parameters. In addition, except MDA, the other indicators showed a significant positive correlation. These results indicated that suitable border irrigation can delay leaf senescence, increase photosynthetic accumulation, and promote grain filling.

## Discussion

### Effect of different border irrigation on antioxidant capacity of flag leaves

The antioxidant system of wheat plants can eliminate the negative effects caused by the accumulation of ROS during leaf senescence. Among them, SOD can catalyze the conversion of O^2−^ into H_2_O_2_, and catalase can remove H_2_O_2_ (Kitonyo et al., 2018). Leaf senescence is affected by several factors, such as temperature, nitrogen fertilizer, and soil water content, of which soil water content is an important factor (Zhao et al., 2008; Saeedipour and Moradi., 2011). Previous studies have shown that flag leaf senescence is closely related to soil water content, and drought or excessive water content can lead to reduced leaf antioxidant enzyme activity (Singh et al., 2012; Kant et al., 2015). In this study, the SOD activity and soluble protein content of flag leaves were significantly reduced under the L20 and L30 treatments due to water stress after anthesis. Adequate water after anthesis in the L40 and L50 treatments is beneficial to maintain high leaf antioxidant capacity and delay leaf senescence (**Figure 2**). In the 2018–2019 growing season, due to higher rainfall after irrigation during anthesis, the SOD activity and soluble protein content of flag leaves in the L50 treatment were lower than in the L40 treatment from 7 to 21 DAA (**Figures 1**, **2**). This is consistent with the conclusion of Kant et al. (2015) that excessive water will reduce the activity of antioxidant enzymes. The MDA content is an important indicator of the degree of lipid peroxidation of the cell membrane. The higher the content of MDA, the more serious the oxidative damage to leaves (Lv et al., 2017). In our study, the MDA content of flag leaves under the L40 and L50 treatments was significantly lower than that of the other treatments from 14 to 28 DAA, which was more conducive to delaying leaf senescence.

### Effect of different border irrigation on fluorescence characteristics and grain filling

Irrational irrigation will increase the production of reactive oxygen species, destroy photosynthetic pigments and lipids, and reduce chlorophyll content (Araki et al., 2012; Kasim et al., 2013). Water stress or waterlogging can reduce the chlorophyll content, Fv/Fm, and ΦPSII, thereby reducing the accumulation of photosynthetic products (Ebrahimiyan et al., 2013; Majidi et al., 2015). Zhang et al. (2015) showed that when the soil water content was 70% field capacity, the SPAD value of flag leaves in anthesis was the highest, which was 19.36% and 4.22% higher than in the 40% and 90% field capacity, respectively. In this study, compared with other treatments, the soil water content of the L40 treatment was more suitable, with higher antioxidant enzyme activity and higher SPAD value of flag leaves after anthesis (**Figure 3**). This explains the significantly higher values of Fv/Fm, ΦPSII, and Qp of the L40 treatment compared with the L30 and L20 treatments from 14 to 28 DAA. Compared with L40 treatment, the fluorescence property and SPAD value of L50 treatment with higher irrigation amount did not significantly increase. This is consistent with the finding of Zhang et al. (2022) that irrigation at anthesis, combined with rainfall events after anthesis, may increase soil water content and reduce chlorophyll content in flag leaves at grain filling, thereby reducing dry matter accumulation.

SPS can convert photosynthetic products into sucrose, which is essential for promoting wheat grain filling and increasing grain yield (Liu et al., 2020a). Studies have shown that soil water content can significantly affect SPS activity in wheat flag leaves (Yang et al., 2004; Wang et al., 2013). Under water stress, the decrease in SPS activity leads to a decrease in sucrose content in leaves, which affects grain filling and grain weight of wheat (Xie et al., 2016; Lv et al., 2017). However, water stress increases the transportation of nutrients and accelerates grain filling (Singh et al., 2012); this explains why the grain weight of the NI treatment is not significantly lower than that of other irrigation treatments at 7 and 14 DAA (**Figure 6**). In the middle and late anthesis stage, sucrose content and SPS activity of flag leaves were significantly reduced in L20 and L30 treatments due to water stress, resulting in a lower grain filling rate (Vmax and Vmean), which was closely related to grain weight (**Tables 4**, **5**). This indicated that optimized irrigation promoted the accumulation of photosynthetic products and maintained higher grain filling after anthesis (Xu et al., 2018). However, the grain-filling rate and grain weight of the L50 treatment with more irrigation were slightly lower than those of the L40 treatment. The reasons may be as follows: (1) higher irrigation and rainfall lead to nitrogen leaching in the root zone and a decrease in the nitrogen content of leaves, thus affecting chlorophyll content and fluorescence characteristics (Liu et al., 2018; Li et al., 2019b), and (2) wheat in the L50 treatment was greedy and late maturing. The high temperature at the later stage led to the rapid maturity of wheat and a decrease in the carbohydrates transferred from leaves and stems to grains (Wang et al., 2016).

### Effect of different border irrigation on grain yield and water productivity

Grain yield is determined by the number of spikes, the grain number per spike, and TGW. Coordinating the relationship among them is crucial to improving grain yield (Lu et al., 2021). For example, high density will lead to increased interspecific competition and reduced grain number per spike and TGW (Liu et al., 2021). The current increase in wheat grain production is mainly through increasing the TGW (Agami et al., 2018). In this study, there was no significant difference in the number of spikes and grain number per spike in the irrigation treatments. The grain yield of the L40 and L50 treatments was significantly higher than that of the L30 and L20 treatments, mainly due to their higher TGW, which was consistent with the conclusion of Agami et al. (2018) (**Table 3**). The TGW is mainly determined by grain-filling rate and grain-filling cycle (Ahmed et al., 2019). In this study, we found no difference in the grain-filling cycle between different treatments; however, the L20 and L30 treatments showed a decreased grain-filling rate due to water stress, resulting in a significant decrease in grain yield and WP. Compared with the L40 treatment, the L50 treatment did not increase TGW and grain yield, and WP was significantly lower than that of the L40 treatment due to the increase in irrigation amount. This was consistent with the findings of Yan et al. (2021), who reported that excessive irrigation not only failed to increase crop yield but also caused resource waste, soil salinity, and reduced soil fertility.

The ultimate goal of agricultural irrigation is to maximize production per unit of crop water consumption (Zhang et al., 2017). Therefore, sprinkler and drip irrigation are better irrigation methods than border irrigation (Li et al., 2019a; Cao et al., 2022). However, since the HPC is mainly cultivated by small farmers and the degree of intensification is low, border irrigation is still the primary method of irrigation at present (Liu et al., 2013). In the future, with higher government investment, the construction of water-saving supporting facilities, and the improvement of farmers’ comprehensive ability, sprinkler and drip irrigation will gradually replace border irrigation. Therefore, this study provides a simple and low-cost water-saving irrigation method for the transition period to sprinkler and drip irrigation in this region, which is of great significance for mitigating the rapid decline of groundwater. In addition, different soil types have a significant impact on the water infiltration rate; therefore, our next step will be to further refine the optimal border length under different soil types to better optimize traditional irrigation.

## Conclusion

In this 2-year field experiment (conducted in light loam soil), compared with other border irrigation treatments, 40 m was more suitable as a high yield and water-saving irrigation border. Water stress (L20, L30) reduced the antioxidant capacity of leaves to varying degrees, thereby reducing the SPAD value and chlorophyll fluorescence parameters, resulting in decreased grain yield and WP. Moreover, the L50 treatment, which had been provided more irrigation, showed no significant increase in various physiological indicators compared with the L40 treatment, resulting in a significant decrease in WP. Therefore, optimizing border length irrigation can reduce the input of irrigation water under the premise of ensuring yield, which is of great significance for the sustainable development of wheat.

## Data availability statement

The original contributions presented in the study are included in the article/**Supplementary Material.** Further inquiries can be directed to the corresponding author.

## Author contributions

Data curation, FY. Formal analyses, FY. Founding acquisition, YS. Investigation, FY and YS. Project administration, YS. Writing original draft, FY. Writing review and editing, YS and ZY. All authors contributed to the article and approved the submitted version.

## Supplementary material

Supplementary Table 1 Soil water content (%) in different soil layer after irrigation under different treatmentsDifferent letters indicate significant statistical differences between treatments (P< 0.05).
