# Supplementary material for: Optimized border irrigation delays winter wheat flag leaf senescence and promotes grain filling
Source: Front Plant Sci. 2023 Feb 13;14:1051323. doi: 10.3389/fpls.2023.1051323 (PMC9968879; doi:10.3389/fpls.2023.1051323)
Supplement: Supplementary Table 1 — Soil water content (%) in different soil layer after irrigation under different treatmentsDifferent letters indicate significant statistical differences between treatments (P< 0.05). [file Table_1.doc]

Table S1 Soil water content (%) in different soil layer after irrigation under different treatments

| Year | Treatment | Jointing | | | |  | Anthesis | | | |
| --- | --- | --- | --- | --- | --- | --- | --- | --- | --- | --- |
|  |  | Before irrigation | | 3 days after irrigation | |  | Before irrigation | | 3 days after irrigation | |
|  |  | 0-40 cm | 0-80 cm | 0-40 cm | 0-80 cm |  | 0-40 cm | 0-80 cm | 0-40 cm | 0-80 cm |
| 2017-2018 | NI | 42.34 | 55.83 | 40.86d | 54.16d |  | 35.25c | 44.81d | 33.36d | 43.25d |
|  | L20 | 42.34 | 55.83 | 66.35c | 65.23c |  | 37.72b | 50.95c | 67.84c | 63.25c |
|  | L30 | 42.34 | 55.83 | 71.50b | 69.84b |  | 39.97a | 52.47b | 71.19b | 68.54b |
|  | L40 | 42.34 | 55.83 | 75.60a | 72.21a |  | 40.54a | 55.58a | 75.04a | 72.61a |
| 2018-2019 | NI | 44.31 | 59.57 | 43.28d | 58.33d |  | 37.85b | 47.59c | 36.09d | 46.32d |
|  | L30 | 44.31 | 59.57 | 71.18c | 69.57c |  | 41.93a | 52.25b | 68.62c | 65.44c |
|  | L40 | 44.31 | 59.57 | 75.58b | 73.46b |  | 42.55a | 56.15a | 73.99b | 72.56b |
|  | L50 | 44.31 | 59.57 | 81.08a | 80.63a |  | 43.19a | 57.18a | 82.19a | 80.42a |

Different letters indicate significant statistical differences between treatments (P< 0.05).
